# Network pharmacology and experimental verification reveal the mechanism of safranal against glioblastoma (GBM)

**DOI:** 10.3389/fonc.2023.1255164

**Published:** 2023-09-05

**Authors:** Xiaobing Yang, Di Lu, Yanfei Sun, Tiandi Wei, Dulegeqi Man, Anbin Chen, Tao Luo, Feihu Zhao, Xuemeng Liu, Bo Cheng, Xu Wang, Peng Zhao, Donghai Wang, Xingang Li

**Affiliations:** ^1^ Department of Neurosurgery, Qilu Hospital, Cheeloo College of Medicine and Institute of Brain and Brain-Inspired Science, Shandong University, Jinan, China; ^2^ Jinan Microecological Biomedicine Shandong Laboratory and Shandong Key Laboratory of Brain Function Remodeling, Jinan, China; ^3^ State Key Laboratory of Microbial Technology, Microbial Technology, Institute, Shandong University, Qingdao, China; ^4^ International Mongolia Hospital of Inner Mongolia, Hohhot, China; ^5^ Department of Neurosurgery, Xinhua Hospital Affiliated to Shanghai Jiaotong University School of Medicine, Shanghai, China; ^6^ School of Basic Medical Sciences, Guizhou Medical University, Guiyang, China; ^7^ Department of Emergency, The Affiliated Hospital of Guizhou Medical University, Guizhou Medical University, Guiyang, China

**Keywords:** glioma, traditional tibetan medicine, safranal, Akt, network pharmacology

## Abstract

**Introduction:**

Safranal is an active component of the traditional Tibetan medicine (TTM) saffron, which has potential anticancer activity.

**Methods and results:**

Here, we studied the therapeutic effect and mechanism of safranal on GBM. CCK-8, GBM-brain organoid coculture experiments and 3D tumour spheroid invasion assays showed that safranal inhibited GBM cell proliferation and invasion *in vitro*. Network pharmacology, RNA-seq, molecular docking analysis, western blotting, apoptosis, and cell cycle assays predicted and verified that safranal could promote GBM cell apoptosis and G2/M phase arrest and inhibit the PI3K/AKT/mTOR axis. *In vivo* experiments showed that safranal could inhibit GBM cell growth alone and in combination with TMZ.

**Conclusion:**

This study revealed that safranal inhibits GBM cell growth *in vivo* and *in vitro*, promotes GBM cell apoptosis and G2/M phase arrest, inhibits the PI3K/AKT/mTOR axis and cooperate with TMZ.

## Introduction

Gliomas represent 81% of malignant brain tumours; they have a 5-year relative survival of 5% and are the most common and aggressive primary brain tumour (1, 2). Despite aggressive treatment protocols, including surgery, radiation, and chemotherapy, there are no known measures to prevent the development of gliomas (3). Therefore, new treatment measures and methods are urgently needed.

Disease is not only caused by a genetic abnormality, but reflects the complex network within or between cells (4). Since the success rate of drug discovery is declining. The “one disease-one target-one drug” doctrine has been challenged (5). Network-based method shows potential clinical application value (4).

Traditional Tibetan medicine (TTM) has a history of thousands of years, and many TTMs have been proven to have anticancer properties (6–10). Saffron is a traditional Tibetan medicine that has been reported to have anticancer effects in previous studies (11–17). The main ingredients of saffron include crocin, crocetin, picrocrocin and safranal (18). We noticed that the molecular weight of safranal is very low, and it was previously reported in the literature that safranal could relieve various nervous system disorders by systemic administration *in vivo*, which indicates that safranal could easily enter the nervous system (19–21). In addition, we found that many articles reported that safranal has anticancer effects (22–26). However, the effect of safranal on glioma, especially glioblastoma (GBM), has not yet been reported.

**Figure 1:**
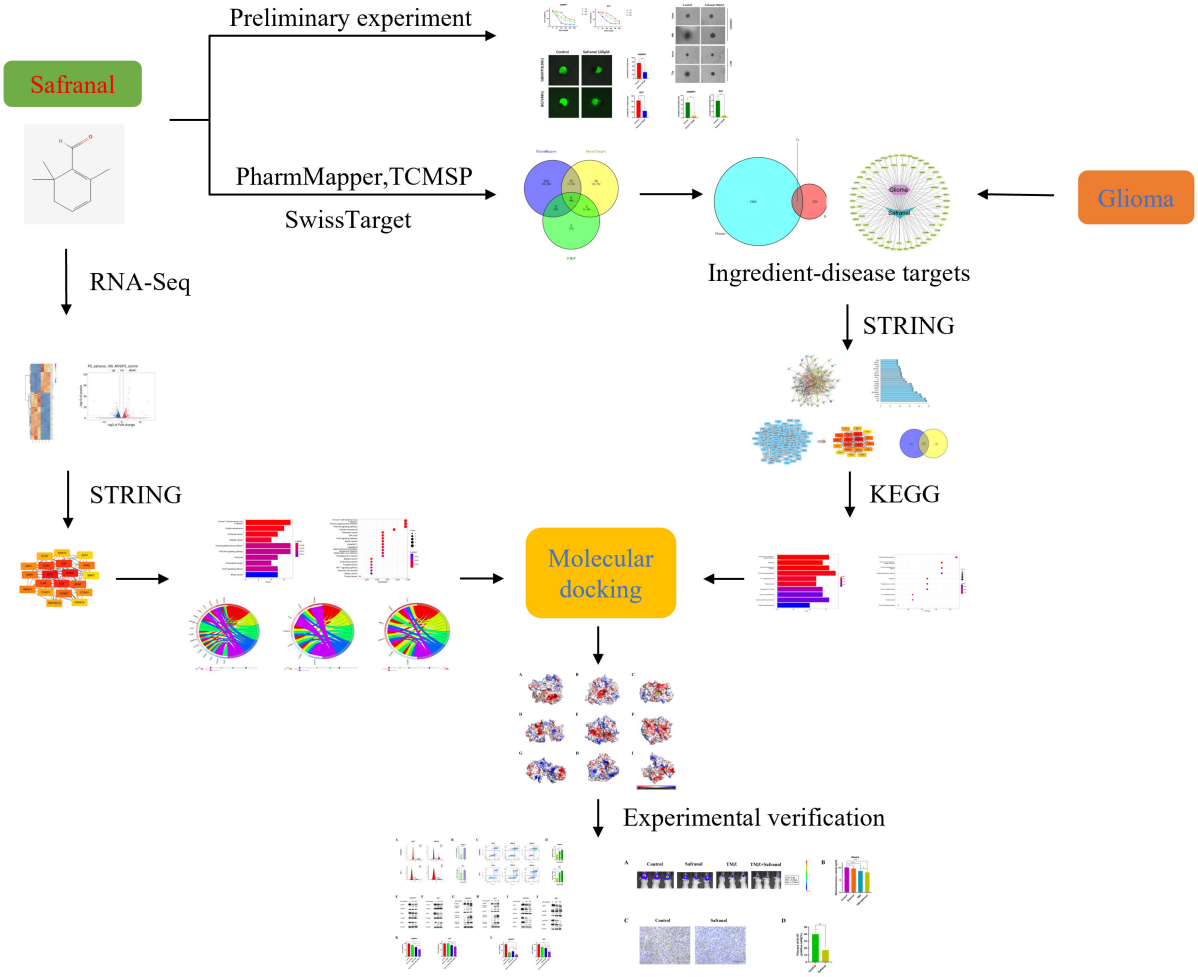
The flow chart of this study.

In this study, CCK-8 and many other *in vitro* experiments were used to preliminarily verify the inhibitory effect of safranal on primary GBM cells. Then, we used network pharmacological methods and RNA-seq analysis to predict the possible safranal pathway in glioma. Finally, western blotting, flow cytometry and other experiments were used to verify the mechanism of safranal, and *in vivo* experiments were used to further verify its effect. In addition, we used *in vivo* and *in vitro* experiments to verify the effect of safranal combined with temozolomide (TMZ). The methodology of this research is summarized in the flowchart (**Figure 1**).

## Materials and methods

### Ethics statement

All animal procedures were approved by the Ethics Committee of Qilu Hospital of Shandong University (permit number: DWLL-2021-095). This study was conducted in full compliance with relevant 100 regulations and guidelines.

### Cell culture

Primary GBM#P3 cells and BG7 glioma stem cells (GSCs) were kindly gifted by Prof. Rolf Bjerkvig (University of Bergen, Bergen, Norway). The cells were cultured in NeurobasalTM Medium (Gibco/Thermo Fisher Scientific; Waltham, MA) supplemented with 2% B27 supplement (Invitrogen; Carlsbad, CA), 2% L-glutamine (BE17-605E, BioNordika; Oslo, Norway), EGF (20 ng/mL; Thermo Fisher Scientific), and basic FGF (10 ng/mL; Thermo Fisher Scientific).

### Chemical and reagents

Safranal (purity ≥98.0%, CAS# 116-26-7) was purchased from MedChemExpress (MCE; Monmouth, NJ,120 USA). It was dissolved in dimethyl sulfoxide (DMSO; Sigma–Aldrich; St. Louis, MO, USA) as a stock solution (1 M) and stored at -20°C. Temozolomide was purchased from Topscience (Shanghai, China). It was dissolved in DMSO as a stock solution (100 mM) and stored at -20°C.

The following antibodies were used: GAPDH (#5174S, CST; Boston, MA, USA), CDK1 (#28439S, CST), CCNB1 (67686-1-Ig, ProteinTech; Wuhan, China), CDK6 (66278-1-Ig, ProteinTech), PARP1 (66520-1-Ig, ProteinTech), Caspase3 (# 14220S, CST), Caspase7 (# 12827S, CST), AKT (# 4691S, CST), mTOR (66888-1-Ig, ProteinTech), phospho-mTOR (Ser2448) (# 5536S, ProteinTech), beta-actin (66009-1-Ig, ProteinTech), Ki67 (GB111499, Servicebio; Wuhan, China)

### Cell proliferation assay

We used Cell Counting Kit-8 (CCK-8; Dojindo, Kumamoto, Japan) to assess cell viability. First, we pretreated the 96-well plate with 1× polylysine (Solarbio) for 15 min. Then, we discarded polylysine, washed the 96-well plate with sterile phosphate buffered saline (PBS) three times and seeded 1×10^4^ cells into each well. After 24 h, the supernatant was replaced with different concentrations of safranal, temozolomide, or vehicle control (dimethyl sulfoxide (DMSO)). At 12, 24, or 48 hours after treatment, GBM cells were incubated with 10 μl of CCK-8 reagent in 100 μl of NeurobasalTM Medium supplemented with 2% B27 supplement, 2% L-glutamine, EGF (20 ng/mL), and basic FGF (10 ng/mL) at 37°C for 1 hour. Then, we used an EnSight Multimode Plate Reader (PerkinElmer; Singapore) to measure the absorbance at 450 nm.

### Cell invasion in 3D culture

GBM#P3 or BG7 cells were seeded into 3D Culture Qualified 96-well spheroid formation plates (Trevigen) with 50 μl of medium, with 3000 cells per well. Then, they were cultured at 37°C and 5% CO2 for 72 hours in a humidified incubator for 72 h to form tumour spheroids. Then, we placed the plate on ice for 15 minutes and added 50 μl of invasion matrix (Trevigen, 3500-096-03) into each well. The plates were centrifuged at 300×g at 4°C for 15 minutes and incubated at 37°C for 1 hour. Then, 100 μl medium with 100 μM safranal or DMSO was added to each well. After 48 h (GBM#P3) or 72 h (BG7), we used a 4× objective to capture the spheroids under a bright field microscope. The images were analysed with ImageJ software.

### GBM-brain organoid coculture assay

The GBM-brain organoid coculture invasion *ex vivo* system was performed as described in a previous study (27). GFP-transfected GBM#P3 cells (3000 cells per well) or BG7 cells (5000 cells per well) were cultured to generate glioma spheroids. Then, mature brain organoids were cocultured for 36 h with GBM#P3 cells or 48 h with BG7 cells. Confocal microscopy (Leica TCS SP8; Wetzlar, Germany) was used to capture GBM cell invasion images. The images were analysed with ImageJ software.

### Target prediction of safranal

We used the Traditional Chinese Medicine Systems Pharmacology (TCMSP) (https://old.tcmsp-e.com/tcmsp.php) database, SwissTargetPrediction (http://www.swisstargetprediction.ch/) database, and PharmMapper (http://www.lilab-ecust.cn/pharmmapper/) database to predict potential targets of safranal. A Venn diagram was made on the Venny website (https://bioinfogp.cnb.csic.es/tools/venny/).

### Determination of potential glioma-related targets

We used the keyword “glioma” to determine the potential glioma-related targets from the GeneCards (https://www.genecards.org/) and OMIM (https://omim.org/) websites.

### Determination of overlapping glioma-related genes and target genes of safranal and construction of the “Drug-targets-disease” network involved in the antiglioma effect of safranal

The “venndiagram” package of R software (version 4.1.3 (2022-03-10)) was used to obtain 72 intersecting genes among glioma-related genes and the safranal target genes, and a Venn diagram was drawn. We used Cytoscape 3.9.1 software to visualize them as a “Drug-Targets-Disease” network.

### Construction and analysis of the protein−protein interaction (PPI) network

The STRING database (https://cn.string-db.org/) was used to obtain the relationships between the 72 intersecting genes. PPIs with a combined score greater than 0.4 were selected and used to construct a PPI network. Then, we used the MNC algorithm in the cytohubba plug-in in Cytoscape 3.9.1 software to find the key proteins in the network.

### GO and KEGG pathway enrichment analysis

The “clusterProfiler” and “org.hs.eg.db” packages in R software were used to analyse the enrichment of drug-disease genes in GO terms and KEGG pathways. Then, we visualized the results with R software.

### RNA-Seq and bioinformatics analysis

We treated GBM#P3 cells with safranal (100 μM) or vehicle (DMSO) for 24 h and performed RNA sequencing (LC-Bio; Shanghai, China). Full data accompanying this experiment are available in the Sequence Read Archive (SRA). The accession number is PRJNA991989. Differentially expressed genes were analysed and visualized by R software. The processes for PPI network analysis and GO and KEGG pathway enrichment analysis were the same as above.

### Molecular docking

We first searched the UniProt ID of the target protein from the UniProt database (https://www.UniProt.org/), and then searched the structure of target proteins in the PDB database (https://www.rcsb.org/) using the UniProt ID of the protein. Protein structures were downloaded. PyMOL software were used to remove water and organic.

The 2D structures of small-molecule ligands were acquired with PubChem (https://pubchem.ncbi.nlm.nih.gov/).The pubchem CID of safranal was 61041, and the molecular weight was 150.22g/mol.

We used AutoDockTools-1.5.7 to add hydrogens to the protein and simulate potential targets for molecular docking. Then, AutoDockVina were used to perform molecular docking.

### Western blotting

Total protein of GBM#P3 or BG7 cells was extracted with cold RIPA Lysis Buffer (Beyotime; Shanghai, China) supplemented with phenylmethanesulfonyl fluoride (PMSF, Beyotime). Western blotting was performed according to previously described protocols (28). We used a chemiluminescence imager (Bio-Rad; Hercules, CA, USA) to detect the chemiluminescent signals according to the manufacturer’s protocol.

### Cell cycle

Seventy percent ethanol was used to dehydrate the cells overnight at 4°C. Then, the cells were stained with PI/RNase (550825, BD Biosciences; San Jose, CA). After 15 min in the dark, the cells were analysed by flow cytometry (Accuri C6, BD Biosciences).

### Apoptosis

We resuspended cells in 300 μL binding buffer and stained them with Annexin V-FITC (BD Biosciences) according to the manufacturer’s instructions. Then, the cells were analysed by flow cytometry.

### Orthotopic xenograft model

For statistical purposes, three nude mice were used in each group. A total of 1×10^6^ luciferase-stable GBM#P3 cells were injected into the brains of 15 4-week-old male nude mice. One week later, tumours were examined using bioluminescence imaging (PerkinElmer IVIS Spectrum; Waltham, MA, USA). We found that in the 15 mice, one had no intracranial tumor formation and two had spinal metastases, so we excluded them. Our inclusion criteria included successful tumor implantation, uniform tumor size, and no spinal metastasis. Exclusion criteria included that there was no intracranial tumor formation, the tumor volume was significantly larger or smaller than that in other mice, and spinal metastasis. Then, we classified the remaining 12 mice as numbers 1-12, used excel software to generate random numbers, arranged the random numbers in reverse order, and then divided the mice into four groups(each group has 3 mice) from top to bottom: control group (n=3), safranal group (n=3), TMZ group (n=3) and TMZ+safranal group (n=3). Tumour volume was monitored using bioluminescence imaging. Mice were euthanized after 2 weeks of treatment.Cages were placed in the same house, keeping the external environment consistent.

### Immunohistochemistry

Mice in each group were harvested, and the brains were removed, fixed in 4% formalin, and prepared as paraffin-embedded sections. Immunohistochemistry was performed as previously reported (28).

## Results

### Safranal suppresses the proliferation and invasion of GBM cells

**Figure 2:**
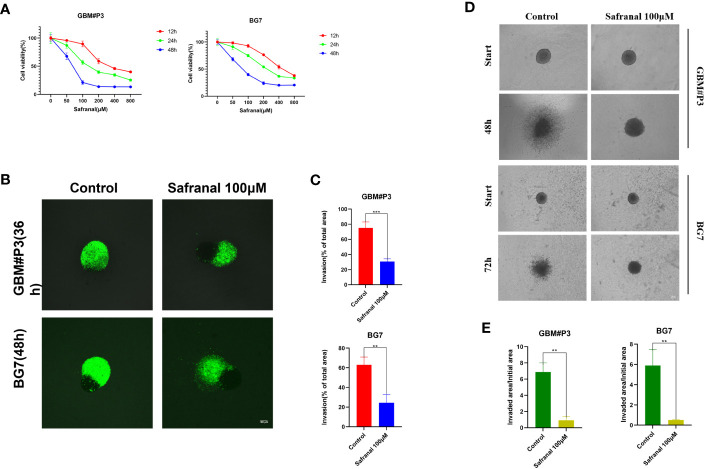
**(A)** CCK-8 assay for cell viability of GBM#P3 and BG7 after treatment with DMSO or different concentrations of safranal. **(B)** Representative images of coculture invasion assays for GBM#P3 and BG7 cells treated with DMSO or 100 μM safranal (scale bar: 100 μm). **(C)** Quantification of the invasion area determined using the percentage of the area invaded after 36 h or 48 h. *P < 0.05, **P < 0.01 and ***P < 0.001. **(D)** 3D invasion assays for GBM#P3 and BG7 (scale bar: 100 μm) treated with safranal (100 μM) or DMSO. **(E)** Quantification of the invaded area/initial area by ImageJ software. *P < 0.05, **P < 0.01 and ***P < 0.001.

**Figure 3:**
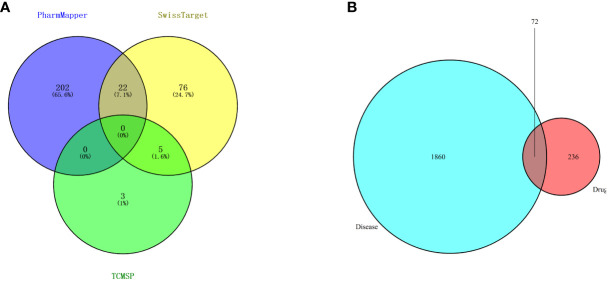
**(A)** Venn diagram of drug targets in the PharmMapper, SwissTargetPrediction and TCMSP databases. **(B)** Venn diagram of drug- and disease-related targets.

First, we performed *in vitro* experiments to test whether safranal had any effect on the proliferation and invasion of primary GBM cells. The CCK-8 assay showed that the viability of GBM cells decreased significantly with increasing safranal concentration (**Figure 2A**). GBM-brain organoid coculture experiments (**Figures 2B, C**) and 3D tumour spheroid invasion assays (**Figures 2D, E**) demonstrated that safranal could inhibit the invasion of GBM cells.

### Determination of targets of safranal

Then, candidate targets of safranal were searched from the SwissTargetPrediction database (http://www.swisstargetprediction.ch/), PharmMapper (http://www.lilab-ecust.cn/pharmmapper/), and TCMSP (https://old.tcmsp-e.com/tcmsp.php) (**Figure 3A**).

### Determination of glioma-related targets

A total of 1932 glioma-related targets were obtained from the GeneCards (https://www.genecards.org/) and OMIM (https://omim.org/) databases.

### Drug–disease intersection targets

**Figure 4:**
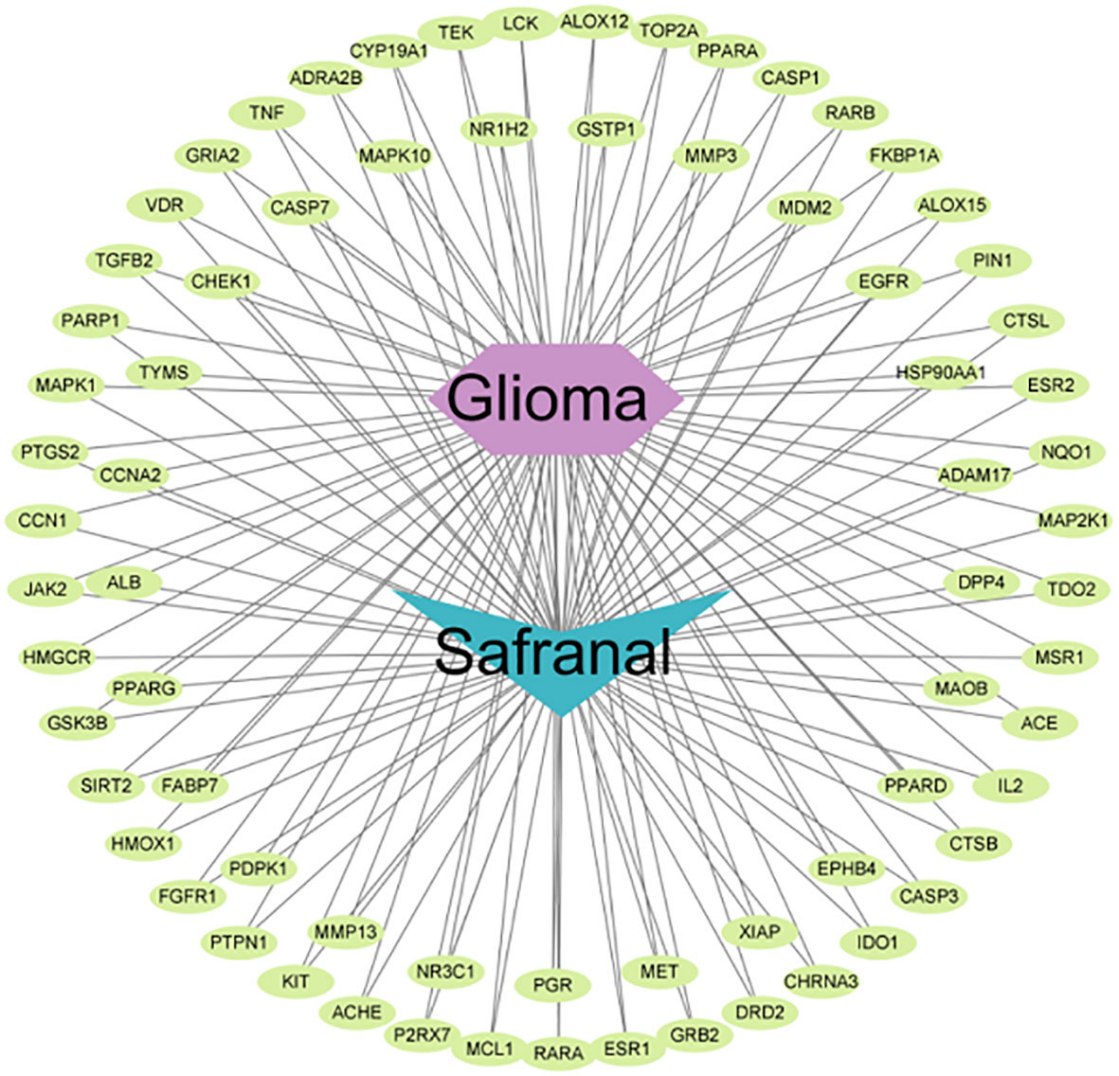
“Drug–targets–disease” network of safranal against glioma.

We used 308 targets of safranal and 1932 glioma-related targets for Venn analysis (**Figure 3B**) to obtain 72 intersecting targets and a drug–targets–disease network (**Figure 4**). The hexagon in violet represents glioma, the “V” in blue represents safranal, and the ellipses in green represent the intersection targets.

### PPI network analysis

**Figure 5:**
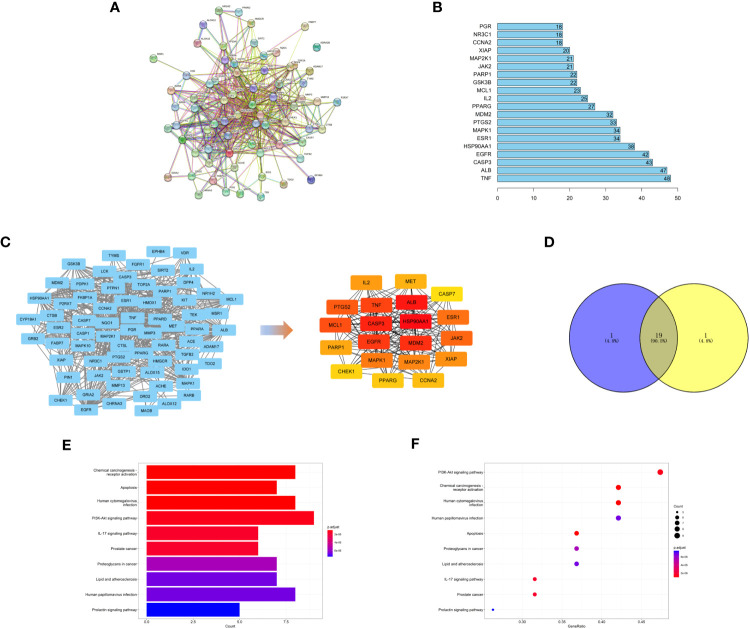
**(A)** PPI network of targets generated using STRING 11.5. Nodes represent proteins. Edges represent PPIs. **(B)** Numbers of edges of 20 core genes in the network. **(C)** Topological analysis of the protein−protein interaction network. **(D)** The top 20 targets with the highest score obtained by the MNC algorithm have 19 intersections with the 20 targets calculated in **Figure 5B**. **(E, F)** KEGG pathway enrichment analysis.

We used the STRING database to find the protein interaction relationship of 72 intersection targets (**Figure 5A**) and counted the top 20 nodes connected most in the network (**Figure 5B**). Then, we imported the results of the STRING database into Cytoscape software and used the MNC algorithm in the CytoHubba plug-in to obtain the top 20 core targets (**Figure 5C**). We found that the top 20 core targets obtained by the MNC algorithm had 19 of the same targets as the top 20 targets calculated in **Figure 5B** (**Figure 5D**), which illustrated that these 19 targets played a key role in the network.

### KEGG pathway enrichment analysis

To explore the possible mechanism of safranal in treating glioma, KEGG pathway enrichment analysis was performed on 19 core targets (**Figures 5E, F**). We found that these 19 targets were enriched in some critical tumour-related pathways, such as apoptosis and the PI3K-Akt signaling pathway. This suggests that safranal may inhibit the growth of glioma cells through these two signaling pathways.

### Analysis of mRNA sequencing data

**Figure 6:**
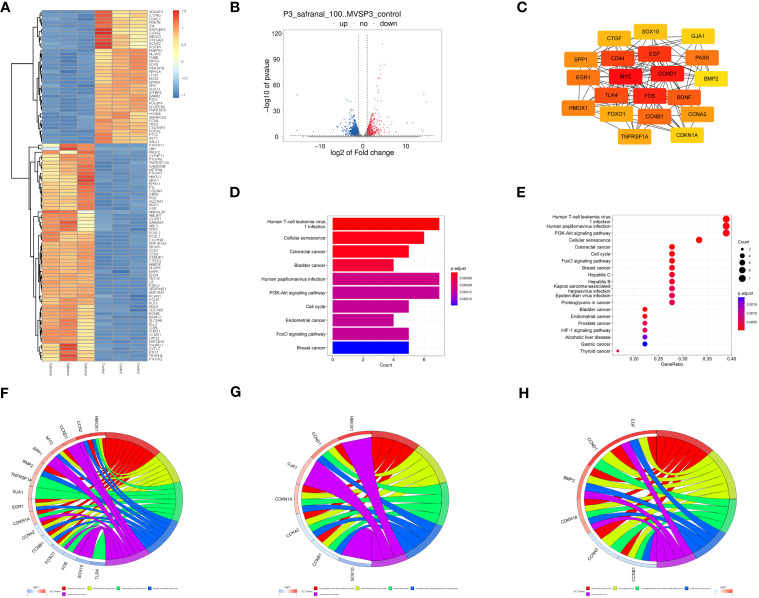
Analysis of mRNA sequencing data. **(A)** Heatmap showing differences in gene expression between the control and safranal-treated groups. **(B)** Volcano plot exhibiting differentially expressed genes between safranal-treated cells and controls. **(C)** Core proteins of the network of proteins coded by differentially expressed genes. **(D, E)** KEGG pathway enrichment analysis of core proteins. **(F–H)** Enriched terms in the biological process (BP), cellular component (CC), and molecular function (MF) categories according to GO enrichment analysis.

Then, primary GBM cells (GBM#P3) were treated with DMSO or 100 μM safranal, and mRNA sequencing was performed. We obtained 1751 differentially expressed genes between the treatment groups (**Figures 6A, B**). We used the STRING database to obtain the interaction network between the proteins encoded by these genes. The MNC algorithm in the CytoHubba plug-in of Cytoscape software was used to find the core protein of the network (**Figure 6C**). Subsequently, KEGG pathway enrichment analysis was performed based on these proteins. We found that these targets were enriched in the cell cycle and PI3K-Akt signaling pathways (**Figures 6D, E**). We continued to perform Gene Ontology (GO) functional analysis. The results showed that the core proteins of the differentially expressed genes were enriched in the cyclin-dependent protein kinase holoenzyme complex, cyclin-dependent protein serine/threonine kinase regulator activity and other cell cycle-related pathways (**Figures 6F–H**).

### Molecular docking study

**Figure 7:**
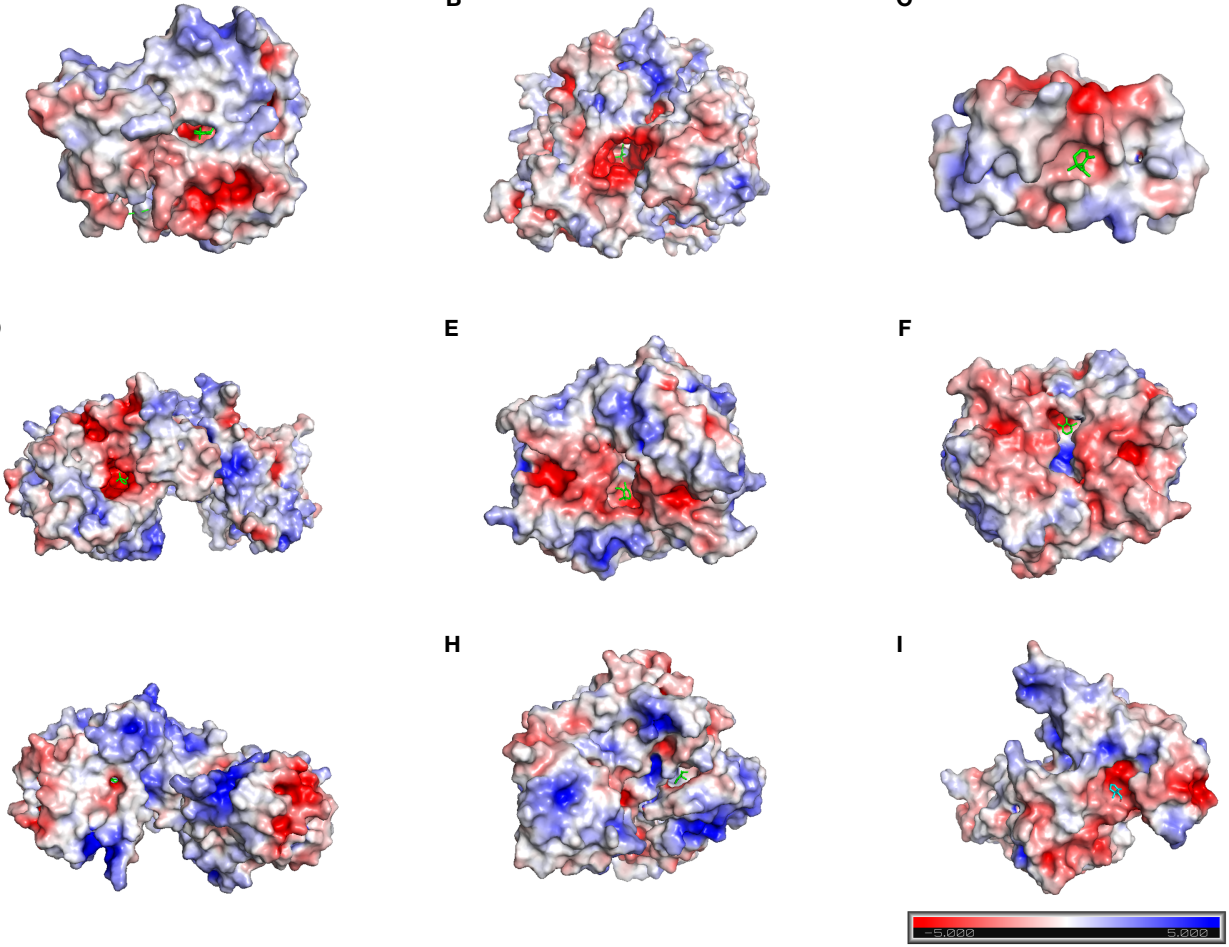
Molecular docking. **(A)** AKT1 with safranal. BE= -6.4 kcal/mol. **(B)** PI3K with safranal. BE= -5.7 kcal/mol. **(C)** mTOR with safranal. BE = -6.4 kcal/mol. **(D)** PARP1 with safranal. BE= -5.8 kcal/mol. **(E)** CASP3 with safranal. BE= -5.3 kcal/mol. **(F)** CASP7 with safranal. BE= -5.6 kcal/mol. **(G)** CCNB1 with safranal. BE= -6.6 kcal/mol. **(H)** CDK1 with safranal. BE= -6.8 kcal/mol. **(I)** CDK6 with safranal. BE=-5.3 kcal/mol. BE, binding energy. Red on the surface of the protein represents a negative charge, while blue represents a positive charge.

Based on the above analysis, we found that the inhibitory effect of safranal on glioma was most likely achieved through apoptosis, the PI3K-Akt signaling pathway and the cell cycle pathway. Therefore, we selected several key proteins in these pathways to perform molecular docking with safranal. As previously reported, safranal can inhibit the PI3K/Akt/mTOR axis, and we docked safranal with the proteins in this axis. The results showed that the binding energies of safranal with AKT1, PI3K and mTOR in the PI3K/AKT/mTOR axis; PARP1, CASP3, and CASP7 in the apoptosis pathway; and CCNB1, CDK1 and CDK6 in the cell cycle pathway were lower than -5 kcal/mol (**Figure 7**, **Table 1**). This indicates that safranal’s inhibitory effect on glioma is most likely to be achieved by inhibiting the PI3K/Akt/mTOR axis, acting on apoptosis-related proteins to increase tumour cell apoptosis, and acting on cycle-related proteins to regulate the tumour cell cycle.

**Table 1:** The docking affinity of safranal binding to key targets.

Protein	Compound	Binding energy(kcal/mol)
AKT1	Safranal	-6.4
mTOR	Safranal	-6.4
PI3K	Safranal	-5.7
CASP3	Safranal	-5.3
CASP7	Safranal	-5.6
PARP1	Safranal	-5.8
CCNB1	Safranal	-6.6
CDK1	Safranal	-6.8
CDK6	Safranal	-5.3

### *In vitro* experiments verified that safranal promotes the G2/M phase arrest of GBM cells

**Figure 8:**
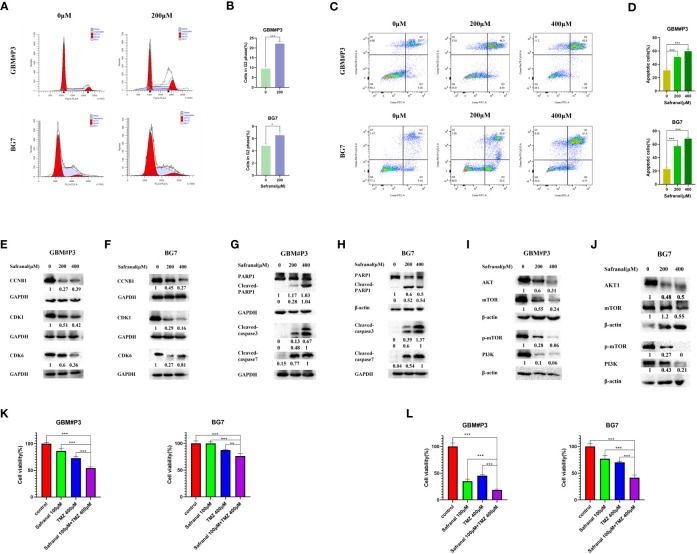
**(A)** Flow cytometry showing cell cycle parameters in GBM#P3 and BG7 cells following safranal treatment for 24 h. **(B)** Graphs representing the proportions of cells in the G2 phase of the cell cycle. **(C)** Flow cytometry showing Annexin V-FITC staining to detect apoptosis in GBM#P3 and BG7 cells treated with safranal or DMSO for 24 h. **(D)** Graphs representing quantification of Annexin V-FITC positive staining for treated cells and controls. **(E, F)** Western blot analysis of cell cycle-related protein markers in GBM#P3 and BG7 cells. **(G, H)** Western blot analysis of apoptosis-related protein markers in GBM#P3 and BG7 cells. **(I, J)** Western blot analysis of protein markers in PI3K/AKT/mTOR axis in GBM#P3 and BG7 cells. **(K, L)** CCK-8 assay for cell viability of GBM#P3 and BG7 after treatment with vehicle (DMSO), safranal, TMZ or safranal combined with TMZ (n = 4) for 24 h **(K)** or 48 h **(L)**.

According to previous studies, safranal could cause G2/M phase arrest of HepG2 cells (23). In our molecular docking prediction, we found that safranal may have a direct interaction with the key proteins CCNB1 and CDK1 of the G2/M transition. We suspect that safranal may cause G2/M phase arrest in GBM cells, so we used the primary GBM cells (GBM#P3 and BG7 cells) for experimental verification. **Figures 8A, B** shows that after treatment with safranal for 24 h, GBM#P3 and BG7 cells showed G2/M phase arrest. In addition, the western blotting results showed that safranal could significantly reduce the protein levels of CDK1, CCNB1, and CDK6 in GBM#P3 and BG7 cells (**Figures 8E, F**).

### *In vitro* experiments verified that safranal promotes the apoptosis of GBM cells

We further assessed the effect of safranal on the apoptosis pathway of GBM cells. In **Figures 8C, D** we used flow cytometry to detect the effect of safranal on the apoptosis rate of GBM#P3 and BG7 cells. As we previously predicted, the apoptosis rate of GBM cells was significantly increased after safranal treatment. The Western blotting results also showed that cleaved caspase3, cleaved caspase7, and cleaved PARP1 were significantly upregulated in GBM#P3 and BG7 treated with safranal (**Figures 8G, H**), which further confirmed that safranal could promote GBM cell apoptosis.

### *In vitro* experiments verified that safranal inhibits the PI3K/AKT/mTOR axis in GBM

In our molecular docking studies, we found that the binding energies (BEs) of safranal with AKT1, PI3K and mTOR were less than -5 kcal/mol, indicating that safranal may be involved in regulating the PI3K/AKT/mTOR axis. This prediction was tested experimentally. The Western blotting results showed that the levels of PI3K, AKT, mTOR and p-mTOR in GBM#P3 and BG7 cells decreased significantly after safranal treatment (**Figures 8I, J**). This confirms that safranal inhibits the PI3K/AKT/mTOR axis in GBM.

### *In vitro* experiments verified that safranal has a synergistic effect with TMZ

TMZ is a first-line treatment for newly diagnosed glioblastoma (29, 30). However, not all patients respond to TMZ (30, 31). It was previously reported that PI3K/AKT axis inhibitors could inhibit temozolomide resistance in glioblastoma cells (32–34), and activation of the PI3K/AKT axis could promote TMZ resistance in GBM cells (35–37). Therefore, we suspect that safranal and TMZ may have a synergistic effect against GBM. We used a CCK-8 experiment for verification. The results in **Figure 8K** and **Figure 8L** show that the combination of safranal and TMZ had a better effect than either single agent alone, which verified that safranal and TMZ could synergistically inhibit the growth of GBM cells *in vitro*.

### *In vivo* experiments verified that safranal could inhibit GBM cell growth and that safranal and TMZ could synergistically inhibit the growth of GBM cells

**Figure 9:**
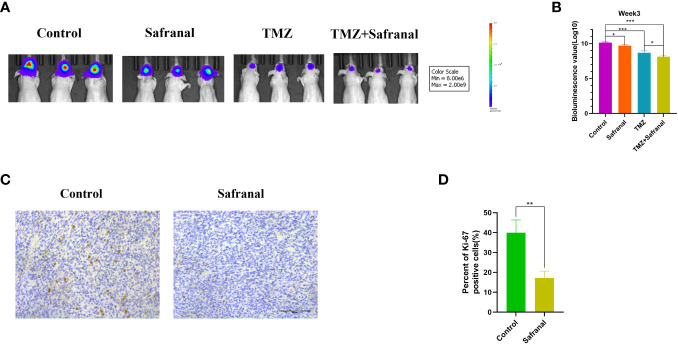
**(A)** Luciferase-stable GBM#P3 cells were orthotopically implanted into nude mice, and tumour growth was followed by the detection of bioluminescent signals under the PerkinElmer IVIS Spectrum at week 3 after implantation. **(B)** Quantification of bioluminescence values to reflect tumour growth at week 3. **(C)** Immunohistochemistry staining for Ki67 in the tumour tissues of nude mice from each group as indicated (scale bars: 50 μm). **(D)** The percentage of Ki-67-positive cells in the tumour tissues.

To further explore whether systemic administration of safranal is effective for intracranial tumours, we implanted 1×10^6^ luciferase-stable GBM#P3 cells into the brains of 4-week-old nude mice. One week later, we randomly divided them into four treatment groups and started treatment. In the control group, the vehicle was injected intraperitoneally (twice a day); in safranal group, intraperitoneal injection of safranal (15 mg/kg, twice daily) was used; the TMZ group received intraperitoneal injection of temozolomide(25 mg/kg) every morning and vehicle every afternoon; TMZ+safranal group received intraperitoneal injection of temozolomide(25 mg/kg) with safranal (15 mg/kg) every morning and safranal 15 mg/kg every afternoon. Two weeks later, tumour growth was evaluated using luciferase bioluminescence. We found that the tumours in the safranal-treated group were significantly smaller than those in the control group, while the intracranial tumours in the safranal combined with temozolomide group were significantly smaller than those in the temozolomide monotherapy group (**Figures 9A, B**).The immunohistochemical results showed that the expression of Ki-67 in tumour cells of safranal-treated mice was significantly lower than that tumour cells of the control mice (**Figures 9C, D**). This indicated that safranal could inhibit the growth of GBM *in vivo*.

## Discussion

Glioma is the most common malignant tumor in the central nervous system, and there is still no effective cure for it. The blood-brain barrier separates the microenvironment of the brain from the blood and is the main physical and physiological barrier that hinders the entry of chemotherapy drugs into the brain. It greatly limits the systemic treatment effect of glioma (38, 39). At present, traditional herbs are becoming increasingly popular in China and other Asian countries. Researchers are using scientific approaches to elucidate their mechanisms, safety and efficacy (40). For example, paclitaxel is a potent anticancer drug that originates from the tree Taxus brevifolia (41); β-Elemene is a sesquiterpene compound extracted from the herb Curcuma Rhizoma, which is used to treat several types of cancer with no reported severe adverse effects (42).

Safranal, one of the most extensively studied bioactive compounds present in herbal medicine saffron (42), could cross brain blood barrier.In previous studies, safranal has been reported to have anticancer effects. However, the effect and mechanism of safranal on GBM are not clear.

Our study reflects the effect of safranal on the growth of GBM cells *in vitro* and *in vivo*. We first used the CCK-8 assay, GBM-brain organoid coculture experiment and 3D tumour spheroid invasion assay *in vitro* to preliminarily find that safranal has an inhibitory effect on the proliferation and invasion of primary GBM cells. Subsequently, we used network pharmacology methods and RNA-seq bioinformatics analysis to predict that safranal may influence the PI3K-AKT signaling pathway, apoptosis, and cell cycle to inhibit glioblastoma growth. In the process of molecular docking verification, we further found that the binding energy between safranal and many key proteins in the PI3K/AKT/mTOR axis, apoptosis pathway and cell cycle pathway was lower than -5 kcal/mol. This shows that safranal’s ability to inhibit GBM cells is most likely achieved by inhibiting the PI3K/Akt/mTOR axis, increasing cancer cell apoptosis and regulating the cancer cell cycle. Subsequently, we used flow cytometry to discover that safranal can induce G2/M phase arrest in GBM cells, and significantly reduce the expression of G2/M transition related proteins CCNB1 and CDK1. The effect of safranal on promoting apoptosis in GBM cells was also verified through flow cytometry and western blot experiments. Additionally, the western blot experiment also confirmed that safranal can inhibit the PI3K/AKT/mTOR pathway in GBM cells.

TMZ is a first-line treatment for GBM and activation of the PI3K/AKT axis can promote temozolomide resistance in glioblastoma (32–34). An increasing in the levels of p-Akt (S473, T308) were found after TMZ treatment in a time- and dose-dependent manner in glioblastoma cell lines, which suggests that the activation of AKT may lead to TMZ resistance (43). Salvianolic acid A treatment could suppress the malignant behaviors of glioma cells and improve TMZ sensitivity through inactivating TAGLN2/PI3K/Akt pathway (32); Bodo Haas at al. also indicated that PI3K pathway seems to play a crucial role in resistance to alkylating agents and might serve as drug target for chemosensitization (33). Therefore, we suspect that safranal may have a synergistic effect with TMZ, which was confirmed by our *in vitro* experiments. Finally, we verified the effect of safranal on GBM and its synergistic effect with TMZ using *in vivo* experiments.

Above all, our study mainly discussed the effect and mechanism of safranal in inhibiting GBM and proved the synergistic effect of safranal and TMZ.

## Conclusion

Safranal inhibited the growth of GBM cells *in vitro* and *in vivo* by promoting GBM cell apoptosis, causing G2/M phase arrest, and inhibiting the PI3K/AKT/mTOR axis. Safranal could cooperate with temozolomide to inhibit GBM cell growth *in vitro* and *in vivo*.

## Data availability statement

The datasets presented in this study can be found in online repositories. The names of the repository/repositories and accession number(s) can be found in the article/supplementary material.

## Ethics statement

The animal study was approved by Ethics Committee of Qilu Hospital of Shandong University (permit number: DWLL-2021-095). The study was conducted in accordance with the local legislation and institutional requirements.

## Author contributions

XY: Investigation, Methodology, Software, Validation, Visualization, Writing – original draft. DL: Investigation, Validation, Writing – review & editing. YS: Investigation, Validation, Writing – review & editing. TW: Investigation, Validation, Writing – review & editing. DM: Investigation, Validation, Writing – review & editing. AC: Investigation, Validation, Writing – review & editing. TL: Investigation, Validation, Writing – review & editing. FZ: Investigation, Validation, Writing – review & editing. XML: Investigation, Validation, Writing – review & editing. BC: Investigation, Validation, Writing – review & editing. XW: Investigation, Validation, Writing – review & editing. PZ: Conceptualization, Investigation, Methodology, Project administration, Supervision, Writing – review & editing. DW: Conceptualization, Methodology, Project administration, Supervision, Writing – review & editing. XGL: Conceptualization, Funding acquisition, Methodology, Project administration, Supervision, Writing – review & editing.
